# Fibrinogen-Based Bioink for Application in Skin Equivalent 3D Bioprinting

**DOI:** 10.3390/jfb14090459

**Published:** 2023-09-05

**Authors:** Aida Cavallo, Tamer Al Kayal, Angelica Mero, Andrea Mezzetta, Lorenzo Guazzelli, Giorgio Soldani, Paola Losi

**Affiliations:** 1 Institute of Life Sciences, Scuola Superiore Sant’Anna, 56127 Pisa, Italy; 2 Institute of Clinical Physiology, National Research Council, 54100 Massa, Italy; 3 Department of Pharmacy, University of Pisa, 56126 Pisa, Italy

**Keywords:** fibrinogen, alginate, bioink, 3D bioprinting, skin equivalent

## Abstract

Three-dimensional bioprinting has emerged as an attractive technology due to its ability to mimic native tissue architecture using different cell types and biomaterials. Nowadays, cell-laden bioink development or skin tissue equivalents are still at an early stage. The aim of the study is to propose a bioink to be used in skin bioprinting based on a blend of fibrinogen and alginate to form a hydrogel by enzymatic polymerization with thrombin and by ionic crosslinking with divalent calcium ions. The biomaterial ink formulation, composed of 30 mg/mL of fibrinogen, 6% of alginate, and 25 mM of CaCl_2_, was characterized in terms of homogeneity, rheological properties, printability, mechanical properties, degradation rate, water uptake, and biocompatibility by the indirect method using L929 mouse fibroblasts. The proposed bioink is a homogeneous blend with a shear thinning behavior, excellent printability, adequate mechanical stiffness, porosity, biodegradability, and water uptake, and it is in vitro biocompatible. The fibrinogen-based bioink was used for the 3D bioprinting of the dermal layer of the skin equivalent. Three different normal human dermal fibroblast (NHDF) densities were tested, and better results in terms of viability, spreading, and proliferation were obtained with 4 × 10^6^ cell/mL. The skin equivalent was bioprinted, adding human keratinocytes (HaCaT) through bioprinting on the top surface of the dermal layer. A skin equivalent stained by live/dead and histological analysis immediately after printing and at days 7 and 14 of culture showed a tissuelike structure with two distinct layers characterized by the presence of viable and proliferating cells. This bioprinted skin equivalent showed a similar native skin architecture, paving the way for its use as a skin substitute for wound healing applications.

## 1. Introduction

Skin is the tissue mostly exposed to pathogens and to the risk of injury [1]. Skin has wound healing ability, but in case of extensive tissue damage or in the presence of other pathologies, such as diabetes, the cell would not be able to repair the wound [2,3]. Nonhealing wounds may require hospitalization, cause disability, and are a portal for local and systemic infectious complications [4]. Conventional approaches are able to reduce morbidity and improve clinical outcomes, but none of the commercial products can restore the normal structure of the skin and its functions [5].

In recent years, a significant improvement in the tissue engineering field was achieved with the introduction of 3D bioprinting technology. This technique, using a layer-by-layer deposition method, enables the biofabrication of living tissue constructs with cells and a biocompatible polymer, bioink. In this way, the precision of traditional tissue engineering technologies is improved [6]. Bioink is a key component of the 3D bioprinting process and of in vitro tissue maturation. The ideal bioink should be printable and should mimic the architectural, biochemical, and biomechanical properties of the extracellular matrix. Despite several advancements, designing the ideal bioink is still a challenge.

Naturally derived hydrogels have been deeply investigated for bioink development [7] and can be divided in biomaterials with high viscosity and printability, such as sodium alginate, chitosan, and methylcellulose and biomaterials able to support cell adhesion, such as collagen, hyaluronic acid, and gelatin [8]. Several bioink formulations based on the combination of these two types of biomaterials are reported in the literature for skin regeneration [5,8].

Among these biomaterials, fibrin has been deeply investigated in the literature as a biopolymer for tissue engineering applications because it possesses remarkable advantages over other biomaterials, which makes it an ideal candidate in skin scaffold fabrication due to its good proangiogenic effects, excellent biocompatibility, and biodegradability, as well as tunable physicochemical features [9,10]. Fibrin, derived by fibrinogen polymerization in the presence of thrombin, has bioactive cues for cell signaling and cell-matrix and cell-cell interactions, mimicking the extracellular matrix. In addition, fibrin can support cell adhesion and growth due to the presence of RGD sequences (Arg-Gly-Asp), which is indispensable for skin cell binding [10]. In addition, for bioprinting applications, fibrinogen, through polymerization in fibrin, allows rapid gelation to maintain the 3D shape of the 3D-printed constructs [11]. However, due to low viscosity and poor shape fidelity, a fibrinogen solution is rarely used on its own as biomaterial in bioprinting [12,13]. Therefore, especially for extrusion-based 3D bioprinting, the addition of other biomaterials, such as alginate, gelatin, and hyaluronic acid, to the fibrinogen solution was considered to obtain a more viscous and printable solution [14]. Ramakrishnan et al. proposed an alginate-gelatin-diethylaminoethyl cellulose-fibrinogen-based bioink for the 3D bioprinting of skin tissue constructs [15]. Xu et al. employed a gelatin/alginate/fibrinogen mixture to assemble adipose-derived stromal cells and complex in vitro 3D models [16]. Han et al. proposed a bioink composed of fibrinogen, gelatin, hyaluronic acid, and glycerol for regenerating patient-specific-shaped and toothlike composite tissue utilizing 3D bioprinting technology [17]. Recently, Budharaju et al. proposed a fibrinogen and alginate blend for cardiac tissue regeneration [18]. Hence, the strategy to combine fibrinogen with a viscous biomaterial that has sol-gel properties was chosen for a novel fibrinogen-based bioink development.

Sodium alginate is a polysaccharide widely used in bioprinting due to its excellent printability and also biocompatibility, biodegradability, viscoelasticity, rapid crosslinking in the presence of calcium ions into a hydrogel, and low cost [19]. Moreover, alginate is also FDA-approved for many biomedical applications and has been used in a number of clinical trials [20]. A stand-alone alginate solution can be printed but with poor resolution, while a semicrosslinked alginate solution obtained by adding Ca^2+^ ions allows the increase in bioink viscosity and, therefore, the improvement of biofabricated structures’ printability [21].

In the present study, a bioink based on a fibrinogen and alginate blend to form a hydrogel by the enzymatic polymerization of fibrinogen with thrombin and by the ionic crosslinking of alginate with divalent calcium ions is proposed for application in skin bioprinting. The fibrinogen- and alginate-based bioink was characterized in terms of physical, mechanical, and biological properties and employed for the 3D bioprinting of a skin equivalent.

## 2. Materials and Methods

### 2.1. Preparation of Fibrinogen-and Alginate-Based Biomaterial Ink

The fibrinogen-based biomaterial ink was prepared by dissolving bovine fibrinogen (65–85% protein, Merck, Darmstadt, Germany) in deionized water to have a final concentration of 30 mg/mL. The solution was mixed using a magnetic stirrer at room temperature for 30 min, then alginate powder (medium viscosity, Merck) was added to obtain a final concentration of 8% (*w*/*v*). The biomaterial ink was semicrosslinked by mixing the fibrinogen-alginate solution with 100 mM CaCl_2_ at volumetric ratios of 25:9 (*v*/*v*). The sterile formulation was obtained using alginate powder prepared according to a previously described protocol [21], sterile fibrinogen, and CaCl_2_ and by performing all procedures under a laminar hood with sterile tools. The semicrosslinked biomaterial ink was centrifuged at 2500 RPM for 5 min to remove air bubbles.

The pH of the final formulation was measured using pH indicator strips.

### 2.2. Biomaterial Ink Homogeneity and Rheological Properties Assessment

The extrusion force, the biomaterial ink filament-like extrusion ability, and therefore the biomaterial ink homogeneity were assessed using a dedicated setup previously reported in [21]. Briefly, if a constant displacement rate is imposed on the syringe plunger, the uniformity of the loaded material would yield a constant extrusion force. Therefore, a syringe, loaded with the proposed formulation and equipped with the same conical nozzle employed during the 3D bioprinting process, was mounted on a dedicated setup to apply a constant displacement rate on the syringe plunger and to measure the extrusion force. The homogeneity was assessed at room temperature.

The rheological properties of fibrinogen-based biomaterial ink were evaluated through rotational tests (shear rate sweep) and oscillatory tests (amplitude sweep and frequency sweep), which were performed as previously described [21]. The rheological characterization was performed at 25 and 37 °C, considered as room temperature and physiological temperature, respectively.

### 2.3. Biomaterial Ink Printability Assessment

Printability is a key property of a bioink that should be flowable or deformable and be able to be deposited precisely with good spatial, temporal, and volumetric control [16]. The printability was evaluated in terms of (a) filament collapse test, (b) spreading ratio, (c) shape fidelity, and (d) printable angle check. A commercial 3D bioprinter, BIO X (Cellink, Gothenburg, Sweden), was employed to perform the printability characterization. The biomaterial ink was loaded into a cartridge compatible with the extrusion printhead, and the temperature was set to 25 °C before starting to print.

The filament collapse test, performed to assess the midspan deflection of a suspended filament of biomaterial ink, was performed according to previously reported works [21,22].

Briefly, immediately after filament printing on a platform with 6 pillars with different gap distances (i.e., 1, 2, 4, 8, and 16 mm), pictures of an extruded filament were closely captured and analyzed using Image J software (NCBI, version 1.54f, Bethesda, MD, USA) for indications of collapse in between the pillars.

**Figure 1:**
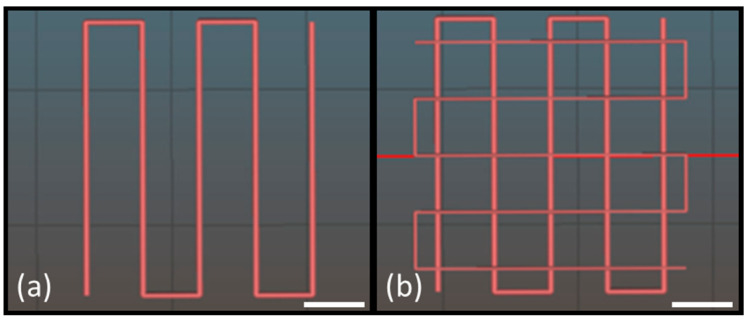
Designed pattern to calculate (**a**) spreading ratio and (**b**) shape fidelity on 4 and 8 overlapped layers; scale bar, 5 mm.

Two different patterns were designed and printed (Figure 1) to calculate the spreading ratio (SR) and the shape fidelity (Pr) according to the following Formulas (1) and (2), respectively:

SR = W_f_/D_n_ (1)

where W_f_ is the width of the printed filament and D_n_ is the nozzle diameter;

Pr = L^2^/(16 × A) (2)

where L is the perimeter and A is the area of interconnected pores.

Moreover, in order to assess the biomaterial ink printing versatility, CAD designs of a circle, a square, and a triangle were made; samples were 3D-printed and observed under a stereomicroscope; and the angles were calculated using Image J.

### 2.4. Mechanical Properties

Three samples (diameter of 8 mm and height of 5 mm) were bioprinted and crosslinked using 50 UT/mL of bovine thrombin in 50 mM CaCl_2_ to investigate the mechanical properties of the proposed biomaterial ink. All mechanical tests were performed using a uniaxial testing machine (ZwickRoell, Ulm, Germany) with a 10 N load cell at 1 mm/min with a maximum displacement of 60%. The linear slope of stress-strain curves was considered to calculate the compressive modulus.

### 2.5. Degradation, Swelling, and Water Uptake Analysis of Biomaterial Ink

To determine the degradation rates over time, the printed and crosslinked samples were weighted (W_o_), then immersed in a complete DMEM culture medium for 14 days, and to simulate culture conditions, samples were maintained at 37 °C and 5% of CO_2_.

The wet weights (W_t_) at 48 h and 3, 7, 10, and 14 days were collected, and the weight reduction was calculated as the difference between the final weight at each time point and the initial weight. The degradation percentage was calculated using the equation reported above (5):

Degradation rate (%) = (W_t_ − W_o_)/W_o_ × 100 (3)

Three samples (diameter of 10 mm and height of 3 mm) were bioprinted and, immediately after crosslinking, freeze-dried for 48 h. The sample weight (W_d_) was measured and then incubated in an RPMI 1640 complete culture medium at 37 °C for 24 h. The excess medium was removed completely, and samples were weighed (W_h_) to calculate the water uptake (WU) and swelling ratio according to the following Formulas (4) and (5), respectively:

WU (%) = (W_h_ − W_d_)/W_h_ × 100 (4)

Swelling = (W_h_ − W_d_)/W_d_ (5)

### 2.6. Morphological Analysis

Crosslinked disks of fibrinogen-based biomaterial ink were fixed in 2.5% glutaraldehyde in 50 mM sodium cacodylate buffer (pH 7.4) for 30 min. The samples were then serially dehydrated for 10 min each in 50%, 70%, 80%, 90%, and 100% ethanol in deionized water for 20 min each in 33%, 67%, and 100% hexamethyldisilazane (HMDS; Merck) in ethanol and in 100% HDMS solution until complete evaporation. Then, samples were sputter-coated with 20 nm gold film. SEM images were taken at an accelerating voltage of 7 kV and 20.0 mm of spot intensity by an electron microscope (FlexSEM 1000, Hitachi, Tokyo, Japan).

### 2.7. Cell Culture

An L929 mouse fibroblast cell line (ICLCATL95001) was obtained from the Interlab Cell Line Collection (Genoa, Italy) and was cultured at 37 °C, 5% CO_2_ in RPMI medium, 10% fetal bovine serum (FBS), 1% penicillin-streptomycin, and 1% glutamine. The medium was changed every 3 days, and the cells were split at 70–80% confluence.

Normal human dermal fibroblasts (NHDF, PromoCell, Heidelberg, Germany) and HaCaT keratinocytes (Istituto Zooprofilattico Sperimentale della Lombardia e dell’Emilia Romagna “Bruno Ubertini”, Brescia, Italy) were cultured at 37 °C, 5% CO_2_ in high-glucose Dulbecco’s Modified Eagle’s Medium, 10% FBS, 1% penicillin–streptomycin, and 1% glutamine. The medium was changed every 3 days, and the cells were split at 70–80% confluence.

All the reagents for cell culture were purchased by Merck.

### 2.8. Evaluation of Biomaterial Ink Biocompatibility

An L929 mouse fibroblast cell line was employed for biocompatibility assessment, and according to ISO 10993, the cytotoxicity assay was performed by an extract assay. Briefly, bioprinted constructs (diameter of 10 mm and height of 0.8 mm) were incubated in culture media for 24 h at 37 °C under stirring with the aim of extracting the cytotoxic substance from the sample. This culture medium was used on an L929 mouse fibroblast cell layer for 24 h at 37 °C and 5% of CO_2_. The viability of the treated cell was evaluated by tetrazolium dye assays and MTT assay, according to the manufacturer’s instructions (CT01-5, Merck). Briefly, 20 μL of an MTT phosphate-buffered solution (0.5 mg/mL) was added to each well, and cultures were incubated at 37 °C for 3 h. The supernatant was removed from the wells by slow aspiration and replaced with dimethylsulfoxide (DMSO, 100 μL per plate) to solubilize the MTT tetrazolium dye. At the end of incubation time, the OD was measured at a wavelength of 550 nm using a microplate reader (SpectraFluor Plus; TECAN Austria GmbH, Grödig, Austria). The incubated medium without a bioink construct was considered as positive control, and the viability assumed as 100%.

### 2.9. 3D Bioprinting of Dermal Layer

NHDF were harvested at 80% of confluence, pelleted (5 min, 1200 RPM), and resuspended in 100 μL of medium. Two Luer-Lock syringes, one loaded with cells and the other one with biomaterial ink, were connected for gentile mixing of cells and bioink. The NHDF-laden bioink was loaded into a cartridge with a piston and a 22 G (0.41 mm as internal diameter) Luer-Lock high-precision conical nozzle. The cartridge was loaded onto the first pneumatic printhead of a BIO X (Cellink, Gothenburg, Sweden) 3D bioprinter. The dermal layer has a diameter of 10 mm and a thickness of 2 mm. The constructs were fabricated using a printhead speed of 10 mm/s and an extrusion pressure of 12 kPa. After printing, the cell-laden constructs were submerged into the crosslinking agent, 50 UT/mL of thrombin in 50 mM CaCl_2_ for 30 min and cultured in a high-glucose DMEM complete medium. The fibroblast’s final concentration was optimized in preliminary experiments testing 1–2–4 × 10^6^ cell/mL.

### 2.10. 3D Bioprinting of Skin Equivalent

The skin equivalent is composed by two different layers, the epidermis containing the HaCaT cell line and the dermal layers characterized by NHDF cells.

NHDF cells were harvested using 1% trypsin, pelleted, and resuspended in 100 µL of medium before bioink addition to have a final concentration of 4 × 10^6^ cell/mL. After printing, the cell-laden constructs were submerged into the crosslinking agent.

After 24 h, HaCaT were harvested at 80% of confluence using 1% trypsin, pelleted, and resuspended in DMEM medium to have a final concentration of 2.5 × 10^6^ cell/mL. On the top part of each sample, 50 μL of cell suspension was bioprinted.

The samples were cultured and submerged into the medium at 37 °C and 5% CO_2_ for 5 days, and then the air-liquid interface (ALI) culture was performed. The culture medium was changed every 3 days. The cell culture medium was adjusted, and the epidermis was exposed to air.

### 2.11. Cell Viability

The cell viability was assessed by qualitative live/dead staining and by quantitative XTT assay.

The live/dead (CBA415, Merck) consists of three selective stains: calcein AM for live cells, propidium iodide for dead cells, and Hoechst 33342 to stain all cell nuclei.

Briefly, samples were washed twice in PBS and incubated in staining solution for 30 min, and the cell viability/morphology was assessed by images randomly taken from constructs observed with a fluorescence microscope (Zeiss Axio Zoom.V16, Carl Zeiss, Oberkochen, Germany) equipped with a Zeiss digital camera (Axiocam 105 color). In particular, the sample images were obtained using a z-stack reconstruction (Zen Blue, Carl Zeiss) at 400 μm as depth range.

The XTT assay was performed immediately after printing and at days 7 and 14 of in vitro culture to quantify the cell viability and, therefore, the cell proliferation. The XTT assay was performed according to the manufacturer’s instructions (Cell Proliferation Kit II, Merck).

### 2.12. Histological Analysis

Histological analysis was performed to evaluate the cell distribution within the 3D structure at days 7, 14, and 21 of culture. Briefly, the 3D-bioprinted constructs were washed two times in PBS for 10 min at 37 °C and fixed in 4% paraformaldehyde solution (PFA) in 50 mM CaCl_2_ for 3 h. Samples were stored in 70% ethanol before paraffin embedding. Samples were dehydrated in ethanol and xylene, transferred in cassettes for paraffin infiltration o.n., and paraffin-embedded. Sections of 7 µm were cut on the microtome (HM350S, Microm, Thermo Fisher Scientific, Waltham, MA, USA) and placed on poly-L-lysine coated slides. Hematoxylin and eosin (H&E) staining was performed.

### 2.13. Statistical Analysis

Data are presented as mean ± standard deviation of a minimum of three replicates in three independent experiments. The statistical significance among the test and the control values (test executed immediately after printing) was determined by an unpaired t-student test, and the values were considered significant at *p* ≤ 0.05. The statistical analysis was performed using StatViewTM 5.0 software (SAS Institute, Cary, NC, USA).

## 3. Results

### 3.1. Biomaterial Ink Homogeneity and Rheological Properties Assessment

**Figure 2:**
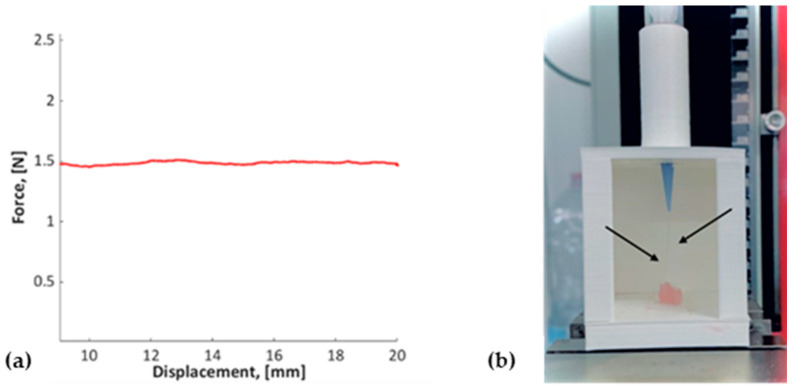
(**a**) Extrusion force measured for the fibrinogen-based biomaterial ink using the dedicated setup implemented to assess the solution homogeneity; (**b**) filament-like shape of extruded biomaterial ink (indicated by black arrows) during the extrusion process.

The extrusion force measured for the proposed biomaterial ink is equal to 1.49 ± 0.04 N (mean ± standard deviation, SD), and the graph of extrusion force versus syringe piston displacement is reported in Figure 2a (the first part of the graph has been discarded to ignore transient resulting from conical nozzle filling). The biomaterial ink can be extruded with a constant force (e.g., low SD value), and therefore, it is a homogeneous solution. Further, this test allowed a qualitative assessment of a biomaterial ink extruded pattern. The biomaterial ink filament-like extrusion is shown in Figure 2b, confirming the free-flowing nature of the proposed formulation.

**Figure 3:**
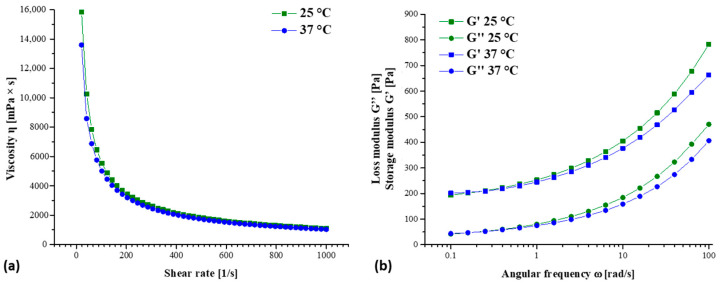
Rheological characterization of biomaterial ink at 25 and 37 °C: (**a**) flow curve as a function of shear rate; (**b**) curves of storage (G′) and loss (G″) modulus vs. angular frequency.

The curves of viscosity vs. shear rate and of storage (G′) and loss (G″) modulus vs. angular frequency at 25 and 37 °C, which represent the standard printing condition and physiological cell temperature, respectively, are reported in Figure 3. For both testing temperatures, the formulation showed decreasing viscosity at an increasing shear rate (Figure 3a); therefore, the bioink is a non-Newtonian fluid with a shear thinning behavior. For an oscillatory test, a frequency sweep test was performed within the linear viscoelastic region of hydrogel to determine the storage and loss modulus of the hydrogel in the angular frequency range of 0.1 to 100.0 rad/s. From the graph in Figure 3b, it is known that the storage modulus (G′) is higher than the loss modulus (G″), revealing the elastic character of bioink and the gel-like properties. Below an angular frequency of 4 rad/s, no significant difference was observed between the storage modulus (G′) and the loss modulus (G″) evaluated at 25 and 37 °C. Instead, an increasing difference was observed above this value, with 25 °C values apparently higher than 37 °C. The most pronounced temperature effect was observed for the storage modulus (G′) compared with the loss modulus (G″).

### 3.2. Biomaterial Ink Printability Assessment

The proposed biomaterial ink was successfully bioprinted with a BIO X 3D bioprinter using a 3 cc cartridge equipped with a 22 G (410 μm diameter) conical nozzle.

**Figure 4:**
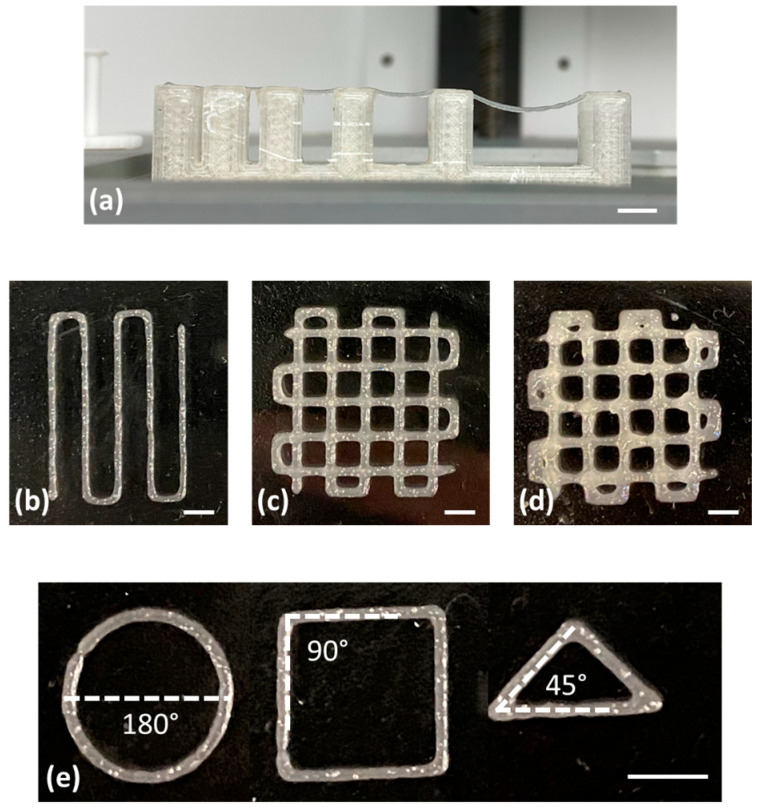
Biomaterial ink printability assessment in terms of (**a**) filament collapse test, (**b**) spreading ratio, shape fidelity, (**c**) 4 layers, (**d**) 8 layers, and (**e**) printable angles; scale bar, 5 mm.

The filament collapse test corresponds to a bioink line printing on a custom platform with defined gaps of 1, 2, 4, 8, and 16 mm. For the proposed formulation, the deflection angle of the printed filament increases with the increase in gap length (Figure 4a). No filament deflection was observed in gap lengths below 4 mm, while the deflection angle was equal to 10° and 28° for gap lengths of 8 and 16, respectively, without the complete filament collapse.

In Figure 4b, the printed pattern for the spreading ratio is shown. The pattern was biofabricated using 7 mm/s as printing speed and 15 kPa as extrusion pressure. The spreading ratio value calculated for fibrinogen-based biomaterial ink is equal to 1.18 ± 0.13 and corresponds to the ratio between the filament width measured by Image J (439 ± 53 µm) and the nozzle diameter, which is 410 µm. The spreading ratio value is nearest to the ideal one (equal to 1), and considering the lower value of SD, the filament edges are smooth and with a uniform finish.

The shape fidelity and the printability of multilayer constructs were assessed by printing squared constructs with grid infill patterns composed of four or eight overlapped layers. The Image J software was employed to measure the perimeter and the area of pores, and then the shape fidelity parameter was calculated (Pr). The Pr value of the fibrinogen-based biomaterial ink is equal to 0.98 ± 0.07 and 0.91 ± 0.08 for four and eight overlapped layers, respectively.

Moreover, samples with circle, square, and triangle shapes were printed, and the angles were measured to assess the matching between the CAD model and printed samples (Figure 4e). The angle match (i.e., 180° for circle, 90° for square, and 45° for triangle samples) confirms the biomaterial ink printing versatility.

### 3.3. Mechanical Properties

**Figure 5:**
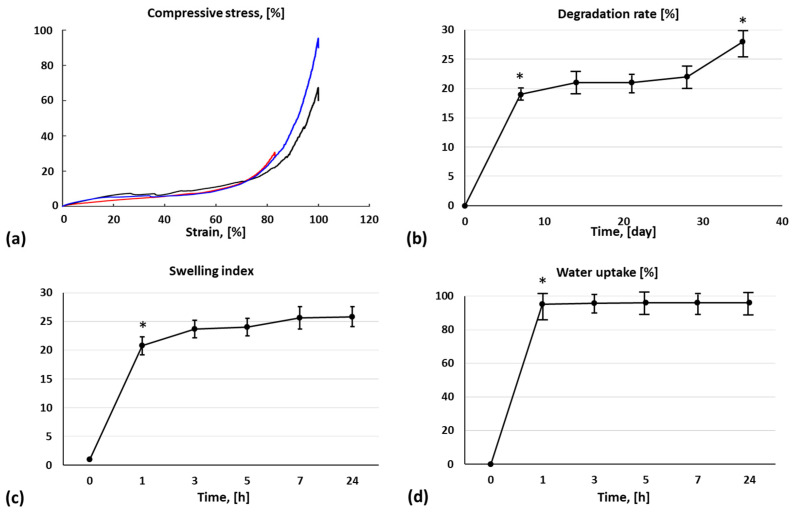
(**a**) Mechanical compression tests on three crosslinked 3D-bioprinted constructs using the fibrinogen-based biomaterial ink, (**b**) degradation rate, (**c**) water uptake, and (**d**) swelling ratio of biomaterial ink; * *p* < 0.05 between two consecutive time points.

The compressive modulus of crosslinked samples of fibrinogen-based biomaterial ink was calculated (Figure 5a) considering the linear slope of the stress–strain curve (10% of strain) before the hydrogel yield’s point. A compressive modulus of 36.5 ± 9.7 kPa was calculated.

### 3.4. Degradation, Swelling, and Water Uptake Analysis of Biomaterial Ink

The biodegradation profile of the 3D-printed constructs (Figure 5b) was assessed by calculating the percentage mass remaining after incubation in a complete cell culture medium for 35 days in standard culture conditions. At day 35, stable and slowly degrading constructs were observed with a remaining mass of 72 ± 4%. In particular, there was a mass loss of about 20% during the first week of incubation, while the sample weight was almost constant during the following 3 weeks, followed by a mass loss increment during the fifth week of incubation.

The swelling index, expressed as a difference in weight between wet and dry samples normalized to the weight of dry samples, and the water uptake, expressed as a difference in weight of wet and dry samples normalized to the weight of wet samples, were evaluated in a dedicated experiment for 24 h, and the results are reported in Figure 5c,d. The swelling index was 20.8 ± 1.6 after 1 h, while water uptake percentage was 95.2 ± 3.6%, confirming that the proposed biomaterial ink is constituted by a high percentage of water.

### 3.5. Morphology Characterization

**Figure 6:**
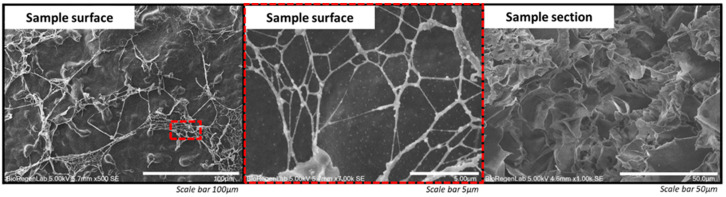
Surface and section SEM images of a crosslinked 3D-bioprinted construct using 50 UT/mL of bovine thrombin in 50 mM CaCl_2_.

The SEM was employed for the morphology analysis of developed fibrinogen-based biomaterial ink after the chemical dehydration of crosslinked samples. In Figure 6, captured images of the surface and section of a 3D-bioprinted construct are reported. As expected, the fibrinogen-based biomaterial ink presents a nanofibrous structure with a thickness of fibrin fibers of 207 ± 48 nm. Fibers create a highly porous microstructure with interconnected pores of variable diameters (range of 20–100 µm), as observed in a sample section.

### 3.6. Biomaterial Ink Biocompatibility Assessment

The biomaterial ink should be able to support in vitro cell adhesion and growth. It should also interact with human tissue in case of in vivo applications. Therefore, the biomaterial ink biocompatibility was evaluated by an indirect method (extract assay). The L929 cell viability was assessed by an MTT assay after 24 h of contact with an extract of biomaterial ink constructs washed or not after crosslinking with 50 UT/mL of thrombin in 50 mM CaCl_2_. The MTT assay results showed a cell viability of 94 ± 4% and 87 ± 6% for samples with and without washing after crosslinking, respectively. Biomaterial ink samples washed after crosslinking showed a lower viability compared with the nonwashed samples without statistical significance. This result is probably related to an excess of thrombin and calcium chloride in the extract medium, which could affect the cell viability for the samples without washing after crosslinking. No cytotoxic effect of biomaterial ink on L929 fibroblasts (cell viability higher than 70% reported by ISO 10993-5) was observed.

### 3.7. Optimization of Cell Density for Dermal Layer 3D Bioprinting

**Figure 7:**
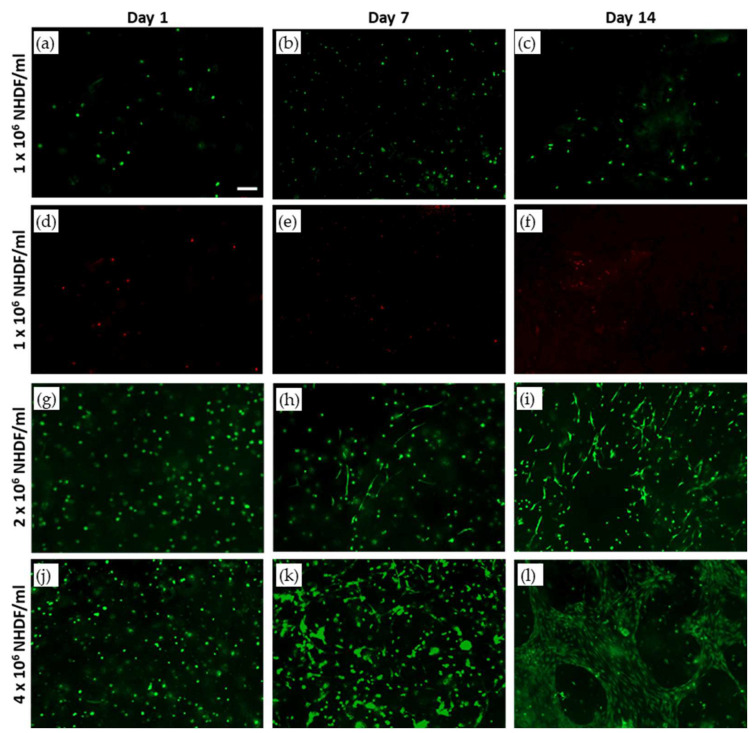
Live/dead staining of 3D-bioprinted samples with cell densities of (**a**–**f**) 1 × 10^6^ NHDF/mL, (**g**–**i**) 2 × 10^6^ NHDF/mL, and (**j**–**l**) 4 × 10^6^ NHDF/mL at days 1, 7, and 14 of in vitro culture; scale bar, 100 µm.

**Figure 8:**
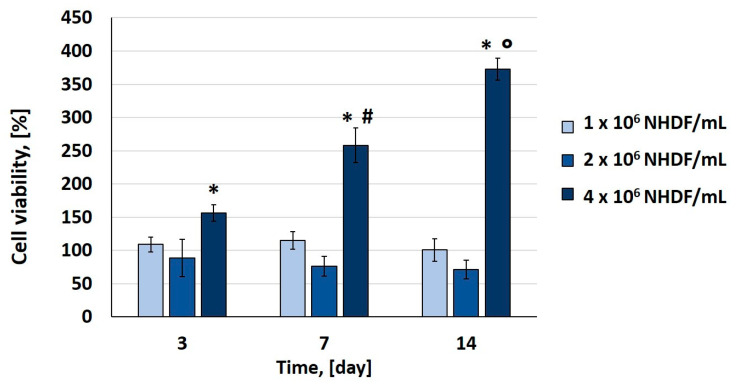
XTT assay on samples biofabricated using 1, 2, and 4 × 10^6^ NHDF/mL at days 0, 3, 7, and 14 of in vitro culture. The absorbance values measured immediately after printing were assumed as 100% of cell viability. Data are presented as mean ± SD (n = 3). * *p* < 0.05 statistically significant differences among the samples biofabricated with 4 × 10^6^ NHDF/mL at days 3, 7, and 14 with respect to day 0; # *p* < 0.05 statistically significant differences among the samples biofabricated with 4 × 10^6^ NHDF/mL at days 3 and 7; **°** *p* < 0.05 statistically significant differences among the samples biofabricated with 4 × 10^6^ NHDF/mL at days 7 and 14.

The live/dead and XTT assays were performed to characterize the viability and the proliferation of NHDF in the 3D-bioprinted dermal layer. Figure 7 shows representative images captured with a z-stack reconstruction at a range depth of 400 μm after live/dead staining on 3D-biofabricated constructs using three different cell densities, 1–2–4 × 10^6^ NHDF cell/mL of bioink. Regarding the samples biofabricated using 1 × 10^6^ NHDF cell/mL, after printing (day 1), a lower number of dead cells (about 25% calculated as the number of dead cells compared with the total number of cells) were observed compared with the living ones. For the samples bioprinted using 1 and 2 × 10^6^ NHDF cell/mL of bioink, cell proliferation within the structure was not observed (Figure 7a–c,g–i). However, in samples with 2 × 10^6^ NHDF cell/mL of bioink, a change in cell morphology was observed. In fact, starting from day 7, it was possible to appreciate a small number of spreading cells in the dermal layer, and at day 14, in different zones of the sample, cell clusters with the fibroblast characteristic morphology were observed (Figure 7h). Therefore, the concentration of cells within the bioink was increased compared with the previous experiments up to 4 × 10^6^ NHDF cell/mL of bioink. Images of live cells within the samples are reported in Figure 7j–l. The cell shape was changed at day 7, and in particular, it was possible to observe excellent cell proliferation. The XTT assay results (Figure 8) are in agreement with live/dead images. A significant increase in cell proliferation rate was observed only in the dermal layer biofabricated with a cell density of 4 × 10**^6^** NHDF cell/mL. In particular, in this case, the measured values of absorbance at days 3, 7, and 14 of culture were statistically different (*p* < 0.05) from the values obtained immediately after printing (assumed as 100%). There was an increase in cell viability up to 250% and 350% at days 7 and 14 of culture, respectively. Considering the results reported above, 4 × 10^6^ cells/mL was the cell density selected for the dermal layer of skin equivalent bioprinting.

### 3.8. Characterization of 3D-Bioprinted Skin Equivalent

**Figure 9:**
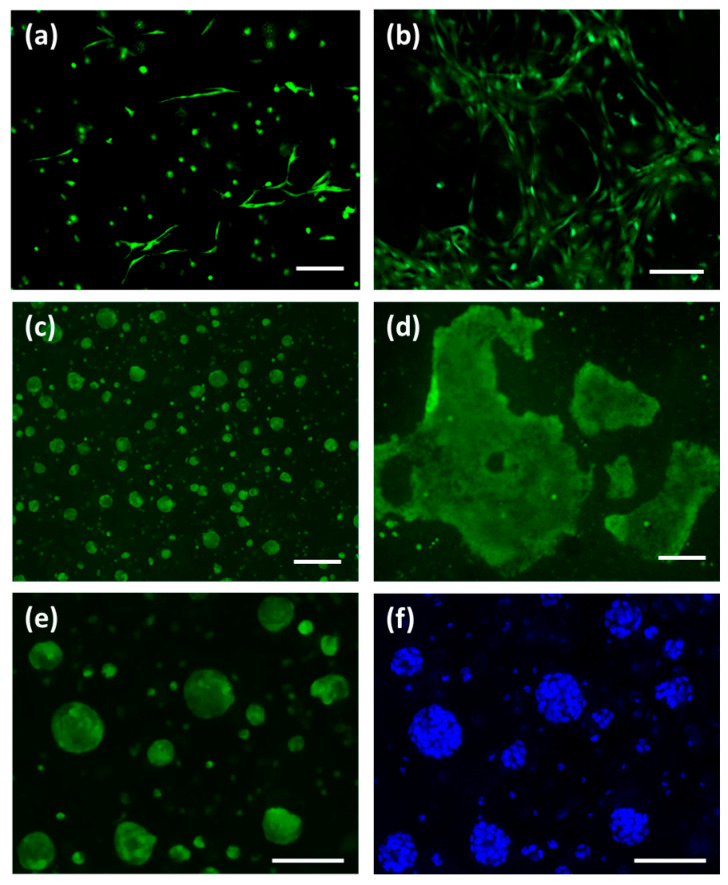
Staining of 3D-bioprinted construct with calcein AM of (**a**,**b**) NHDF cells at days 7 and 14 of in vitro culture and of (**c**,**d**) HaCaT cell at days 7 and 14 of in vitro culture; (**e**,**f**) calcein AM and Hoechst 33342 staining of HaCaT keratinocytes; scale bar, 100 µm.

The 3D-bioprinted skin equivalent with human fibroblasts and keratinocytes was investigated at days 0, 7, and 14 of culture through live/dead cell staining with Hoechst 33342 in addition to identifying the cell nucleus and by histological analysis. Figure 9a,b show NHDF cells after calcein AM staining at days 0, 7, and 14. Fibroblasts were viable, and, in particular, at day 14, compared with day 7, fibroblasts were spreading. The staining of viable keratinocytes with calcein AM and of nuclei with Hoechst 33342 at day 3 is reported in Figure 9e,f. The staining with Hoechst 33342 enables the distinction of single cells that compose each HaCaT cluster. The proliferation of fibroblasts and keratinocytes and the increased spreading of fibroblasts and of cluster dimensions were observed after 7 and 14 days of in vitro culture.

**Figure 10:**
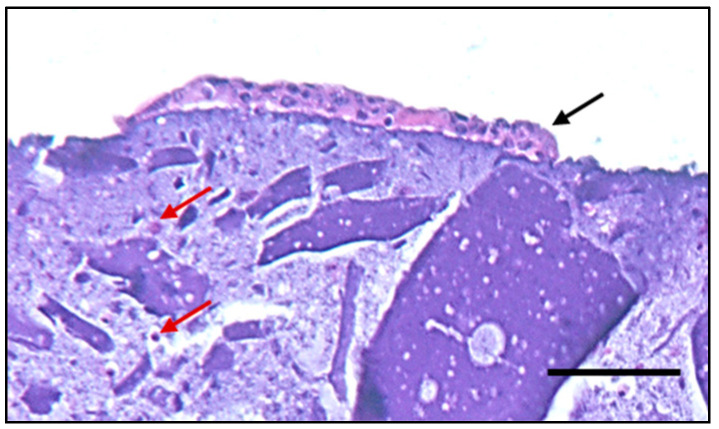
Representative image of H&E staining at day 14 of culture of equivalent fabricated by HaCaT seeding 24 h after dermal layer bioprinting. HaCaT and NHDF cells are indicated by black and red arrows, respectively; scale bar, 200 µm.

The histological section with H&E staining on a sample fixed in PFA at day 14 of culture, shown in Figure 10, confirmed that each colony is composed of a variable number of cells and mono- or multiple layers of keratinocytes as well as the homogenous distribution of NHDF cells within the dermal layer. In addition, a clear stratification of the epidermal layer with keratinocytes and the dermal one with fibroblasts was observed.

## 4. Discussion

The aim of this study was to develop a bioink based on a fibrinogen and alginate blend to obtain a fibrin-based hydrogel by the enzymatic polymerization of fibrinogen with thrombin and by the ionic crosslinking of alginate with divalent calcium ions for skin equivalent 3D bioprinting. The final biomaterial ink formulation is composed of 30 mg/mL of bovine fibrinogen, 6% of alginate, and 25 mM of CaCl_2_ in H_2_O. CaCl_2_ was added to obtain the semicrosslinking of alginate and, in this way, to increase the viscosity of fibrinogen and alginate solution [21]. The proposed formulation has a neutral pH compatible with cell physiology. The crosslinking solution for sol-gel transition after bioprinting is composed of 50 UT/mL of bovine thrombin in 50 mM of CaCl_2_.

The biomaterial ink owns a high concentration of fibrinogen; in this way, stable fibrin hydrogels after sample crosslinking can be obtained [15,16,17].

For successfully bridging the gap from research to applied clinical practice, there are many challenges, such as maintaining cell viability pre-, during, and post-3D bioprinting, but also a reliable and effective sterilization of the applied polymers is critical during the early stage of bioink development [23]. Therefore, sterility was considered as a fundamental condition in the definition of the bioink preparation protocol.

The proposed formulation was characterized considering the homogeneity, the rheological properties, the printability, and mechanical, physical, and biological properties to evaluate if the ideal bioink requirements are met.

The homogeneity of biomaterial ink directly affects the printability and the uniform cell distribution within the construct and, hence, aids in cell viability after extrusion. Considering the instability of the semicrosslinking of alginate using Ca^2+^ ions [24,25], the homogeneity of the final semicrosslinked solution was evaluated to validate the biomaterial ink preparation protocol and, in addition, to assess the biomaterial ink ability to remain homogeneous under a certain shear stress. The implemented custom setup allowed for mimicking the extrusion process that occurs during the 3D bioprinting phase, and therefore, the measured magnitude force represents also the force required to achieve the target constant displacement rate. The force resulted in a constant, suggesting the homogeneity of the solution, and the force magnitude depends on several parameters, such as the displacement rate, material viscosity, and orifice diameter [26]. Further, this test allowed a qualitative assessment of a bioink extruded pattern. The proposed formulation is ideated for extrusion-based bioprinting that requires a filament-like extrusion to build a stable construct with a homogenous distribution of cells within the structure. A smooth fluid flow and continuous extrusion of a hydrogel filament through the nozzle was observed as a clear indication of a good printable formulation, allowing an accurate and controlled filament deposition [27].

A bioink, to be compatible with extrusion-based bioprinting technology, should exhibit a shear thinning behavior [22]; therefore, the rheological properties were investigated. The proposed formulation respected this requirement, displaying a decreasing viscosity when an increasing shear stress was applied and, in addition, showed gel-like properties (G′ > G″). Moreover, the fibrinogen addition did not change the rheological properties of the stand-alone alginate formulation reported in our published work [21]. Higher viscosity and values of the loss and storage moduli were reported for other fibrinogen-based bioinks described in the literature [15,18]. This is related to the different concentrations of fibrinogen, alginate, and calcium chloride selected [18] as well as the presence of other biomaterials, such as the gelatin and diethylaminoethyl cellulose in the bioink formulations [15].

The printing resolution is more complex to be evaluated because it is affected by many factors, such as pressure, feed rate, nozzle shape, and nozzle diameter. The resolution is considered part of the printability evaluation [28]. Therefore, the biomaterial ink was characterized in terms of printability. The printability concept is not univocally defined and, in this study, is considered as the possibility to extrude hydrogel with acceptable accuracy compared with the 3D model. The printability was assessed through filament collapse test, spreading ratio, shape fidelity parameter, and angle matching. The proposed formulation can be printed without filament deformation until a pillar gap of 4 mm. The deformation is caused by gravity and reaches the equilibrium point with force against the deformation caused by the filament Young’s modulus. An ideal bioink should have a filament width equivalent to the nozzle diameter, and therefore, a spreading ratio value of 1.0 represents perfectly smooth and uniform filament printing [29]. The spreading ratio value is nearest to the ideal one, and considering the lower value of SD, the filament edges are smooth and with a uniform finish, as also shown in Figure 4b,c. The obtained value of the spreading ratio is close to values reported as acceptable in the literature, resulting in good printability [30,31,32,33,34]. An ideal axial macroporosity in a 0–90° laydown pattern should thus display a squared (or rectangular, depending on the designed strand-to-strand distances) profile in the x–y plane. In this case, a high geometric accuracy would result in a printability index of Pr = 1 (square shape transversal pore geometry), while Pr < 1 and Pr > 1 correspond to a rounder or irregularly shaped transversal geometry, respectively [29,35]. As expected, the shape fidelity gets worse by increasing the number of bioprinted layers (Figure 4c,d). This is probably related to the slight filament settling at the junction point and to the layer collapsing due to weight and/or gravity factors. However, the proposed biomaterial ink has better printability compared with other fibrinogen-based bioink reported in the literature. For example, Ramakrishnan et al. proposed an alginate–gelatin–diethylaminoethyl cellulose–fibrinogen–based bioink that can be crosslinked using divalent calcium ions. The bioink shape fidelity was not maintained in the three and five overlapped layer structures [15].

The scaffolds used in skin tissue engineering should exhibit adequate and similar mechanical properties of a native tissue extracellular matrix. In particular, the tensile strength should be in the range of 5–30 MPa with elongation at break in the range of 35–115% and Young’s modulus between 10^4^ and 10^6^ Pa [15,36,37,38,39]. Mechanical properties of bioink wherein cells are embedded are an important factor for cell viability and proliferation. For this reason, the compressive modulus of crosslinked samples of fibrinogen-based biomaterial ink was calculated, and the mechanical behavior was comparable with other bioinks for skin tissue engineering or soft tissue bioprinting described in the literature [40,41,42]. Moreover, the fibrin construct can be easily handled without losing its integrity.

In vitro degradation analysis of crosslinked 3D bioprinted constructs shows controlled biodegradation. This allows cells to synthesize and deposit an extracellular matrix, which provides additional stability to the construct. Stability is a key factor for a successful tissue engineering. The proposed fibrinogen-based biomaterial ink is much more stable than the semicrosslinked alginate biomaterial ink [21,43]. The high in vitro stability, a remaining mass of 72% at 35 days, shown by the proposed biomaterial ink could be related to the dual crosslinking approach based on the thrombin and calcium chloride combination effect: enzymatic crosslinking for the fibrinogen and ionic for alginate. The degradation analysis of the hydrogel construct proposed by Ramakrishnan et al. showed a remaining mass without thrombin of nearly 39% by 28 days [15]. According to our results, recently, Budharaju et al. reported in their study the optimization of dual thrombin and calcium chloride crosslinking for a fibrinogen-alginate-based bioink loaded with human ventricular cardiomyocytes demonstrating the advantage of dual crosslinking combined on enzymatic and ionic crosslinking [18]. Probably, the selected fibrinogen concentrations in the proposed biomaterial ink could be responsible for the low degradation rate shown by fibrin constructs. Additionally, Han et al., in their study on the fabrication of a three-dimensional dentin–pulp complex with patient-specific shapes by inducing localized differentiation of human dental pulp stem cells within a single structure, demonstrated that fibrinogen concentration also affected the degradation properties of the bioink [17]. In addition, the effect on degradation rate of cell addition to the biomaterial ink should be considered to investigate the cell role in a sample biodegradation. The construct capability to retain aqueous medium (water absorption), which is necessary for cell growth and wound healing, and a reduced swelling index, which corresponds to the alteration of constructs’ geometrical features, are demonstrated. The water uptake is the cause of sample swelling; therefore, these parameters are directly correlated between them and also with the sample degradation [44]. These results, combined with the degradation analysis, suggest the stability of a construct without excessive swelling for potential applications in in vitro cell culture studies and may provide a suitable environment for in vivo wound healing applications.

The interconnected network of pores observed in the construct by SEM should provide adequate space for cells to grow and allow the media and other nutrients to reach them as required for an ideal bioink [35,45].

Finally, the proposed formulation resulted a biocompatible in vitro experiment against L929 mouse fibroblasts with an indirect method as described in the literature [15].

On the basis of the obtained results, the fibrinogen-based solution proposed in this study can be used as a bioink, and therefore, a cell-laden formulation was employed in the 3D bioprinting process.

Cell density is a critical factor of the bioprinting process because the addition of cells can alter the physical properties of a printable bioink, impeding or inducing cellular sedimentation or affecting the printability and shape fidelity of the final construct. In addition, from a biological point of view, cell density plays an important role in cell–cell signaling and the regulation of cellular differentiation. The optimal cell density for a 3D bioprinting is largely dependent on the type/dimension of cells and also on the 3D matrix in terms of biomaterials employed [46,47]. Human fibroblast (NHDF)–laden bioink with 1, 2, or 4 × 10^6^ cells for mL was used for 3D dermal layer bioprinting. Considering the results of live/dead staining (about 25% of death cells) on a sample biofabricated using 1 × 10^6^ cell/mL, the protocol used for cell encapsulation in fibrinogen-based bioink, with a particular focus on holding time and the printing process performed with an extrusion pressure of 12 kPa, with a printhead speed of 10 mm/s and a 22 G nozzle, does not significantly affect cell viability. Hence, the fibrinogen-based bioink allows human viable fibroblast encapsulation and bioprinting.

Excellent results in terms of cell morphology and proliferation were obtained with 4 × 10^6^ cells/mL. In this case, a significantly higher level of NHDF proliferation was observed in comparison with the other tested cell concentrations, with more than 150% increase following 1 week of culture and 250% increase following 2 weeks of culture compared with viability measured at day 1 after printing (assumed as 100%). The comparison of three different and incremental cell densities confirms that cell density controls’ cell–cell interaction within a 3D-bioprinted cell-laden scaffold and modulates cell morphology [46,47].

The skin equivalent was bioprinted using 4 × 10^6^ fibroblasts for mL of bioink, followed by HaCaT keratinocyte bioprinting. The HaCaT cell density, which corresponds to 1.25 × 10^5^ cell/cm^2^, was selected as the most promising cell density reported in the literature [48]. The samples were cultured in vitro for 14 days, showing fibroblast and keratinocyte proliferation with a tissuelike structure. This result is related to the fibrin in bioink and pore sizes suitable for cell attachment, stimulating spreading and proliferation. In particular, cell spreading in the bioprinted layer was observed since day 7, while in the fibrinogen-based bioink proposed by Ramakrishnan et al., cell spreading was not observed [15]. However, a complete confluence of keratinocyte layers, as observed in H&E staining sections, was not achieved at the end of the culture experiment. An in vitro culture period of 14 days is a short time compared with other studies reported in the literature, in which for a complete epithelial layer differentiation, at least 1 month of in vitro culture is required [48,49]. In addition, after 2 weeks of in vitro culture, the samples can be easily handled (without sample breaking), confirming the slow degradation rate observed in samples without cells and the satisfactory mechanical properties of the bioink for the selected application. The in vitro stability of cell-laden samples could be probably related to the cells’ ECM deposition.

## 5. Conclusions

In this study, a bioink based on a fibrinogen and alginate blend to form a hydrogel by enzymatic polymerization of fibrinogen with thrombin and by the ionic crosslinking of alginate with divalent calcium ions was developed and employed for skin equivalent biofabrication. The characterization of biomaterial ink showed a homogeneous blend with a shear thinning behavior, excellent printability, adequate mechanical stiffness, porosity, biodegradability, and water uptake, and it is in vitro biocompatible. Therefore, it was employed for the skin equivalent biofabrication using human primary fibroblasts (NHDF) and human keratinocytes (HaCaT). Like most of the studies reported in the literature, this study used only dermal and epidermal cells but was missing other skin cell types. Therefore, skin biomimicking has not been fully achieved yet. Nevertheless, this bioprinted skin equivalent showed a similar native skin architecture with two distinct layers, paving the way for its use in skin applications. In addition, to improve the epidermal layer stratification and differentiation, human primary keratinocytes could be used instead of HaCaT cells.

In conclusion, these results suggest the excellent potential of the alginate–fibrinogen blend as bioink for the 3D bioprinting of skin equivalent useful for wound healing applications.

## Data Availability

All data relevant to the publication are included.
